# Privacy-Preserving Process Mining in Healthcare †

**DOI:** 10.3390/ijerph17051612

**Published:** 2020-03-02

**Authors:** Anastasiia Pika, Moe T. Wynn, Stephanus Budiono, Arthur H.M. ter Hofstede, Wil M.P. van der Aalst, Hajo A. Reijers

**Affiliations:** 1 School of Information Systems, Queensland University of Technology, Brisbane 4000, QLD, Australia; m.wynn@qut.edu.au (M.T.W.); sn.budiono@qut.edu.au (S.B.); h.a.reijers@uu.nl (H.A.R.); 2 RWTH Aachen University, Process and Data Science Group, 52062 Aachen, Germany; 3 Utrecht University, Department of Information and Computing Sciences, 3508 TC Utrecht, The Netherlands

**Keywords:** process mining, healthcare process data, data privacy, anonymisation, privacy metadata

## Abstract

Process mining has been successfully applied in the healthcare domain and has helped to uncover various insights for improving healthcare processes. While the benefits of process mining are widely acknowledged, many people rightfully have concerns about irresponsible uses of personal data. Healthcare information systems contain highly sensitive information and healthcare regulations often require protection of data privacy. The need to comply with strict privacy requirements may result in a decreased data utility for analysis. Until recently, data privacy issues did not get much attention in the process mining community; however, several privacy-preserving data transformation techniques have been proposed in the data mining community. Many similarities between data mining and process mining exist, but there are key differences that make privacy-preserving data mining techniques unsuitable to anonymise process data (without adaptations). In this article, we analyse data privacy and utility requirements for healthcare process data and assess the suitability of privacy-preserving data transformation methods to anonymise healthcare data. We demonstrate how some of these anonymisation methods affect various process mining results using three publicly available healthcare event logs. We describe a framework for privacy-preserving process mining that can support healthcare process mining analyses. We also advocate the recording of privacy metadata to capture information about privacy-preserving transformations performed on an event log.

## 1. Introduction

Technological advances in the fields of business intelligence and data science empower organisations to become “data-driven” by applying new techniques to analyse large amounts of data. Process mining is a specialised form of data-driven analytics where process data, collated from different IT systems typically available in organisations, are analysed to uncover the real behaviour and performance of business operations [1]. Process mining was successfully applied in the healthcare domain and helped to uncover insights for improving operational efficiency of healthcare processes and evidence-informed decision making [2,3,4,5,6]. A recent literature review [3] discovered 172 articles which report applications of various process mining techniques in the healthcare domain.

While the potential benefits of data analytics are widely acknowledged, many people have grave concerns about irresponsible use of their data. Healthcare data can include highly sensitive attributes (e.g., patient health outcomes/diagnoses, and the type of treatments being undertaken). Hence, privacy of such data needs to be protected. An increased concern of society with protecting the privacy of personal data is reflected in the growing number of privacy regulations that were recently introduced or updated by governments around the world. These government regulations provide general governance principles for the collection, storage, and use of personal data. For example, the General Data Protection Regulation (GDPR) (https://gdpr-info.eu/) requires that organisations fulfil “the right to be forgotten” (i.e., erase all personal data under certain conditions when requested). Failure to comply with data privacy regulations can lead to significant penalties (e.g., organisations can be fined up to 20 million Euro or 4% of their annual global turnover, whichever is higher, if they breach the GDPR). Data privacy requirements are also often included in legislation which regulates the healthcare sector (e.g., in the Australian Healthcare Identifiers Act 2010 (https://www.legislation.gov.au/Details/C2017C00239)).

The need to comply with strict data privacy requirements often results in a decreased data utility, i.e., the effectiveness of the anonymised data for data analysis. Consider the recently introduced My Health Records Amendment (Strengthening Privacy) Bill 2018 (https://www.myhealthrecord.gov.au/about/legislation-and-governance/summary-privacy-protections). This bill allows Australians to delete their electronic health records at any time. While this allows protecting privacy, the quality and value of data analysis may decrease.

The need to consider data privacy in process mining and develop privacy-aware tools was already raised in the Process Mining Manifesto [7]. However, the process mining community, until recently, largely overlooked the problem. A few recent articles highlight “a clear gap in the research on privacy in the field of process mining” [8] and make first attempts to address some privacy-related challenges (e.g., ref. [8,9,10,11,12]) yet, significant challenges remain.

Privacy considerations are quite well-known in the field of data mining and several privacy-preserving data transformation techniques were proposed [13,14] (e.g., data swapping, generalisation, or noise addition). *Although there are many similarities between data mining and process mining, some key differences exist that make some of the well-known privacy-preserving data mining techniques unsuitable to transform process data.* For example, the addition of noise to a data set may have an unpredictable impact on the accuracy of all kinds of process mining analyses.

In this article, we analyse data privacy and utility requirements for process data typically recorded in the healthcare domain, assess the suitability of privacy-preserving data transformation methods proposed in the data mining and process mining fields to anonymise healthcare process data, and evaluate the impact of selected privacy-preserving methods on process mining results. The results of the analyses and the evaluation showed that the problem of privacy protection for healthcare data while preserving data utility for process mining analyses is challenging. As a possible solution to the problem, we propose a privacy-preserving process mining framework which is based on the use of privacy metadata, and we propose a privacy extension for XES logs.

This journal article presents an extended version of the workshop paper presented at PODS4H 2019 [15] with two new additions (Section 5 and Section 7). Section 5 presents new insights from a detailed evaluation conducted on three healthcare event logs. Section 7 describes the proposed privacy metadata for XES logs. In addition to new research contributions presented in these two sections, the related work discussion in Section 2 has been extensively revised.

This article is organised as follows. We present related work (Section 2), analyse data privacy and utility requirements for healthcare process data (Section 3), and assess the suitability of existing privacy-preserving methods to anonymise healthcare process data (Section 4). We then evaluate the impact of some generic data transformation approaches on the results of various process mining methods applied to three publicly available healthcare event logs (Section 5), describe the proposed privacy-preserving process mining framework in Section 6, and describe the proposed privacy extension in Section 7. Section 8 concludes the paper.

## 2. Related Work

In this section, we first provide an overview of privacy-preserving data mining (Section 2.1) and describe selected generic data transformation approaches (Section 2.1.1) and privacy models (Section 2.1.2). We then discuss existing privacy-preserving approaches proposed by the process mining community (Section 2.2).

### 2.1. Privacy-Preserving Data Mining

Privacy and access control considerations are quite well-known in several research communities, including the statistical community, the database community, the cryptographic community, and the data mining community. Several data transformation techniques, access control mechanisms, and frameworks to preserve data privacy were proposed by these communities [13,14,16,17]. Techniques for protecting respondents’ privacy, which originated in the statistics community, are often referred to as Statistical Disclosure Control (SDC) [17]. The data mining community is concerned with protecting privacy of personal information that may be recorded about individuals (e.g., medical history); methods proposed by this community are usually referred to as Privacy-Preserving Data Mining (PPDM) [13]. Distributed PPDM methods, which aim to protect privacy of multiple data owners who wish to conduct analysis of combined data sets without disclosing their data to other data owners [13], originated in the database and cryptographic communities [17]. Although privacy-preserving methods “have evolved in a fairly independent way within research communities”, many methods proposed in one community are also applied by other research communities [17]. For example, data swapping, suppression, noise addition, and k-anonymity are discussed in both the SDC literature [18] and the PPDM literature [13]. In this article, we use term PPDM to refer to all privacy-preserving methods (regardless of their origin).

To preserve data privacy, privacy-preserving methods usually reduce the representation accuracy of the data [13]. Such data modifications can affect the quality of analysis results. The effectiveness of the transformed data for analyses is often quantified explicitly as its *utility* [13]. The main challenge of privacy-preserving methods is to minimise privacy risks while maximising data utility [13,18,19].

Most privacy-preserving methods aim to minimise risks of identity disclosure or sensitive attribute disclosure [13,19]. Identity disclosure happens when an individual is identified by an attribute (e.g., social security number) or by a combination of attributes (e.g., age, gender, postcode, and job title). Sensitive attribute disclosure happens when a value of some sensitive attribute is discovered by an adversary (e.g., medical diagnosis). Many privacy-preserving data transformation methods that originated in the statistics community (e.g., data swapping or noise addition) do not provide any formal privacy guarantees and “the level of protection is empirically evaluated a posteriori for a specific dataset” [19]. For example, distance-based approaches quantify the level of protection by computing distance between the original data set and the transformed data set [18]. Other PPDM methods can “attain a predefined notion of privacy and offer a priori privacy guarantees over the protected data”, they are usually referred to as privacy models [19]. For example, privacy guarantees can be specified in terms of *k-anonymity*. A data set satisfies k-anonymity if each record in the data set is indistinguishable from at least *k-1* other records. We discuss in detail methods from both categories in Section 2.1.1 and Section 2.1.2.

Methods for measuring data utility either assess information loss by quantifying differences between original and anonymised data [18] or are designed for specific applications; for example, utility can be assessed by comparing classification accuracy [20] or regression coefficients [18] obtained from original and anonymised data. Utility measures designed for specific applications are more informative as different data analysis methods have different data requirements [18,20].

Privacy-preserving data mining techniques can be generic or specific [14]. *Generic* approaches modify data in such a way that “the transformed data can be used as input to perform any data mining task” [14]. These approaches can provide anonymisation (in this article, *anonymisation* refers to any method that can protect data privacy) by modifying records without introducing new values (e.g., data swapping) or they can modify original values (e.g., by adding noise). In specific approaches privacy preservation is tailored for specific data mining algorithms (e.g., a privacy-preserving approach for clustering of big data on cloud [21] or privacy-preserving decision tree classification) [14]. Furthermore, *outputs* of some data mining algorithms can also be sensitive and methods that anonymise such outputs were proposed (e.g., association rule hiding) [13]. Finally, *distributed* privacy-preserving methods are proposed for scenarios in which multiple data owners wish to derive insights from combined data without compromising privacy of their portions of the data [13]. Such methods often use cryptographic protocols for secure multi-party computations (SMC) [13]. In this article, we focus on protecting privacy of process data *within* a healthcare organisation, distributed privacy scenarios are considered outside the scope of this work. Furthermore, we do not analyse specific PPDM methods and methods for protecting output privacy as they are tailored to specific data mining algorithms (and are not applicable to other data or process mining algorithms).

#### 2.1.1. Generic Privacy-Preserving Data Transformation Approaches

In this subsection, we describe generic privacy-preserving data transformation approaches, such as data swapping, noise addition, suppression, generalisation, and micro-aggregation [13,18]. We evaluate the suitability of these approaches to anonymise process data in Section 4.1.

*Data swapping* involves enacting privacy to a dataset by adding a degree of uncertainty. Uncertainty is introduced into individual records by swapping the true values of sensitive attributes between subsets of records [16]. This method allows anonymisation of both numerical and categorical attributes.

*Noise addition* can be used for both numerical and categorical data [14]. Numerical values are often anonymised by factoring randomly and independently generated “white noise” into the original data [13]. White noise is generated using a random distribution, often either uniform or Gaussian. Adding noise to categorical values is more complex, and can be achieved, for example, using clustering-based techniques [22]. This method preserves the aggregate distribution of the attribute values; however, the randomisation leads to the loss of individual records.

*Suppression* anonymises data by omission. Values can be removed under three types of data suppression [13]. The most common type is column suppression which targets the presence of highly sensitive attributes whose values directly identify an individual (e.g., patient names or identification numbers). Alternatively, row suppression is used when outlier records are infrequent and difficult to anonymise. Value suppression omits selected sensitive attribute values.

*Generalisation* methods replace data values with approximate values making it difficult for adversaries to identify records with full confidence [13]. The process of generalising usually includes the construction of a generalisation hierarchy, which is a predefined classification of values at decreasing levels of granularity. For numeric data, values are sorted into numerical ranges. For categorical data, a domain expert creates semantically meaningful generalisations using a tree structure.

*Micro-aggregation* methods consist of two steps: partition and aggregation [23]. Partition organises the original records into clusters whose data is similar to each other. An aggregation operator is then used to compute a collective value (e.g., mean, median, interval, or mode) for each cluster. Original values in each cluster are then replaced with the computed collective value. Micro-aggregation can be applied to both continuous and categorical data without the need for the data author to create generalised categories. Various approaches to perform micro-aggregation were proposed; for example, a hybrid micro-aggregation approach which is “based on fuzzy possibilistic clustering” [24].

#### 2.1.2. Privacy Models

*k-Anonymity* is one of the oldest privacy models whose goal is to prevent identity disclosure [13]. k-Anonymity methods require that “all combinations of key attributes in a database be repeated at least for *k* records” [17]. Many approaches for achieving k-anonymity were proposed and often use suppression, generalisation, or micro-aggregation. k-Anonymity helps to prevent identity disclosure; however, it cannot prevent sensitive attribute disclosure [13]. For example, a dataset may satisfy k-anonymity; however, a group of records with identical key attributes may have the same value of a sensitive attribute (e.g., diagnosis). Although one cannot link an individual to a record, they can still discover the diagnosis.

*l-Diversity* and *t-closeness* privacy models target this shortcoming of k-anonymity [13,17]. *l-Diversity* requires that each group of records with identical key attributes (an equivalence class) contains at least *l* “well-represented” values for a sensitive attribute [13]. There are different definitions of “well-represented” values; for example, *distinct l-diversity* requires that each equivalence class contains at least *l* distinct values [17]. The *t-closeness* model requires that the distance between the distribution of a sensitive attribute in an equivalence class and the distribution of the attribute in the data set does not exceed a threshold *t* [17]. While the t-closeness model provides a better privacy protection than the l-diversity model, it does so at the expense of data utility by “severely impairing the correlations between confidential attributes and key attributes” [17].

The *differential privacy* model “ensures that (almost, and quantifiably) no risk is incurred by joining a statistical database” [25]. In this model, sensitive information is collected by a trusted curator who releases “statistical facts about the underlying population” [26] and original data is not released.

These privacy models were developed for statistical databases in which “records refer to individuals that are described by a set of usually uni-valued attributes” [19]. The assumption that a record contains all information about an individual is not true for process execution data in which personal information can be scattered across multiple records and there could be dependencies between such records (we discuss this in detail in Section 3). Therefore, existing privacy models that are focused on statistical databases are not directly applicable to process execution data.

### 2.2. Privacy-Preserving Process Mining

Several articles made first attempts to address some privacy-related process mining challenges [8,9,10,11,12,27,28,29,30]. Mannhardt et al. [8] analysed privacy challenges in human-centered industrial environments and provided some generic guidelines for privacy in process mining. Michael et al. [29] proposed a privacy system design for process mining which considers a number of “privacy elements”; for example, what information should be collected, by whom, for what purposes, how it is stored, and who can access it. Rafiei and van der Aalst [31] proposed a log anonymisation method which protects resource information “against frequency-based attacks” and allows discovering roles from the anonymised log. Liu et al. [11] presented a privacy-preserving cross-organisation process discovery framework based on access control. Tillem et al. [27,28] presented interactive two-party protocols for discovery of process models from encrypted data, which are based on multiple communication rounds (and have high computation costs).

The first privacy-preserving data transformation approach presented in the process mining community [9] proposes to use deterministic encryption methods for anonymisation of event log attribute values. (Such methods are also a part of the confidentiality framework proposed by Rafiei et al. [12].) Timestamps are treated as numeric values and are encrypted in a way that preserves the order of events. Deterministic encryption methods produce “the same ciphertext for a given plaintext” and preserve differences between values, which is important for process mining [12]. Encryption only provides weak data privacy protection and “could be prone to advanced de-anonymization techniques” [9].

More advanced privacy-preserving process mining approaches proposed by Rafiei et al. [12], Fahrenkrog-Peterse et al. [10], and Mannhardt et al. [30] will be discussed in detail in Section 4.

Unlike these privacy-preserving process mining approaches, in this article we focus on privacy of healthcare process data, evaluate the suitability of existing privacy-preserving data transformation approaches to anonymise such data, and propose a privacy-preserving process mining framework, which is based on the use of privacy metadata.

## 3. Data Privacy and Utility Requirements: Healthcare

In this section, we first describe process execution data typically recorded in the healthcare domain (Section 3.1) and legislative requirements that regulate the use of such data (Section 3.2). To realise our objective of privacy-preserving process mining for the healthcare domain, we then analyse privacy requirements for healthcare process data (Section 3.3), which is followed by a discussion of data requirements of process mining approaches to analyse healthcare processes (Section 3.4).

### 3.1. Healthcare Process Data

Process mining uses process data in the form of an event log, which represents collated and aggregated data from IT systems available in organisations. An event log contains events where each event refers to a case, an activity, a point in time, transaction type (e.g., *start* or *complete*), and (optionally) a resource and data attributes. An event log can be seen as a collection of cases and each case can be seen as a sequence of events.

**Figure 1:**
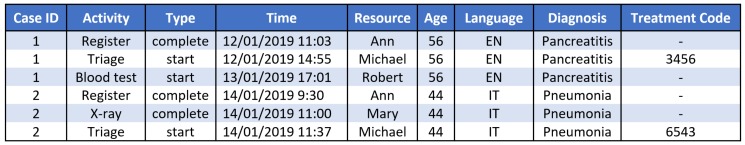
Example of an event log with typical healthcare data attributes.

Cases in healthcare processes typically refer to patients receiving treatments (e.g., a patient’s pathway) and resources refer to medical personnel involved in the process. Figure 1 depicts an example event log which contains six events (represented by rows) related to two cases (*1* and *2*). For example, we can see that case *1* refers to a patient whose age is *56*, who speaks English and was diagnosed with pancreatitis; activity *Register* is completed in this case; activity *Blood test* was started on *13/01/2019* at *17:01* by *Robert*; and treatment code *3456* is associated with activity *Triage* in case *1*. Data attributes can refer to cases (e.g., age, language, and diagnosis) or to events (e.g., treatment codes are recorded for events associated with activity *Triage*). In this example, we used some data attributes which are recorded in two publicly available healthcare logs. The healthcare MIMIC data set (https://mimic.physionet.org/mimicdata/) contains information about language and diagnosis (as well as ethnicity, religion, marital status, and insurance). The Dutch academic hospital event log (https://data.4tu.nl/repository/uuid:d9769f3d-0ab0-4fb8-803b-0d1120ffcf54) contains information about age, diagnosis, and treatment codes.

### 3.2. Legislative Requirements

An increased concern of people with protecting the privacy of their data is reflected in the growing number of privacy regulations that were recently introduced (e.g., the EU General Data Protection Regulation (GDPR) 2018, the California Consumer Privacy Act of 2018) or updated by governments around the world (e.g., Australian Privacy Regulation 2013 under the Privacy Act 1988). In addition, data privacy requirements are often included in legislation governing specific sectors, e.g., Australian Healthcare Identifiers Act 2010.

Guidance for de-identification of protected health information in the US is provided in the Health Insurance Portability and Accountability Act (HIPAA) Privacy Rule. For example, the “safe harbor” de-identification method of the HIPAA Privacy Rule prescribes removal of all elements of dates (except year) related to an individual (e.g., admission or discharge dates) (https://www.hhs.gov/hipaa/for-professionals/privacy/special-topics/de-identification/index.html#protected). In Australia, the Office of the Australian Information Commissioner provides guidelines for the use of health information for research. The guidelines prescribe de-identification of personal information by “removing personal identifiers, such as name, address, d.o.b., or other identifying information” and “removing or altering other information that may allow an individual to be identified, for example, because of a rare characteristic of the individual, or a combination of unique or remarkable characteristics” (https://www.oaic.gov.au/engage-with-us/consultations/health-privacy-guidance/business-resource-collecting-using-and-disclosing-health-information-for-research). Furthermore, the recently introduced My Health Records Amendment (Strengthening Privacy) Bill 2018 (https://www.myhealthrecord.gov.au/about/legislation-and-governance/summary-privacy-protections) allows Australians to opt out of having an electronic health record and allows the deletion of their records permanently at any time. Whilst providing strong privacy protections for Australians, these measures also introduce data quality issues such as missing and incomplete data. This reduces the utility of data, decreases the accuracy of results, and influences analysis.

Privacy of public healthcare data is typically protected by replacing sensitive attribute values with anonymised values (e.g., treatment codes are used in a publicly available Dutch academic hospital event log and subject IDs are used in the healthcare MIMIC data set) or by removing sensitive attributes from data (e.g., employee information is removed from both the Dutch hospital and MIMIC data sets). The former method only provides weak privacy protection while the latter method can significantly decrease data utility.

### 3.3. Privacy Requirements for Healthcare Process Data

Healthcare process data can contain sensitive information such as patient or employee names or identifiers. Other attributes in the event log can also reveal patient or employee identities when combined with background knowledge about the process. For example, accident or admission time, a rare diagnosis or treatment, or a combination of age and language could potentially identify a patient. An employee could be identified by the combination of an activity name and execution time (e.g., when a blood test is always performed by the same employee during a shift). Hence, typical event log attributes such as *case ID, activity, time, resource* and many data attributes (e.g., a patient’s personal and treatment information) can contribute to identity disclosure.

Furthermore, relations between events in a log can contribute to identity disclosure and this is especially pertinent for a healthcare event log due to the high variability of process paths typical for the sector [2]. Consider, for example, the Dutch hospital event log where 82% of cases follow unique process paths. Hence, someone with knowledge of the process could link these cases to individual patients. Moreover, cases which follow the same process path can include other atypical behaviors. In the Dutch hospital log, the fifth most frequent process variant is followed by 8 cases: 7 cases are related to only one organisational group (“Obstetrics and Gynecology clinic”) and only one case is also related to the “Radiotherapy” group. Although the case does not follow a unique process path, the relation to the “Radiotherapy” group is unique and could be used by someone with knowledge of the process to identify the patient. Other examples of *atypical process behaviour* which could contribute to a patient’s identity disclosure include abnormally short or long execution times of activities or cases, or an abnormally low or high number of resources involved in a case. Healthcare processes may contain many different types of atypical process behaviour as these processes are often “complex and highly variable” and involve “frequent interactions between clinicians, nursing staff, diagnostic support specialists and administrative personnel” [2].

If employees can enter or modify some process information (e.g., activity labels), this can introduce additional privacy threats. For example, an activity label may be modified to include a doctor’s name, or some doctor may have a habit to not record some data attribute (hence, missing data can indicate the doctor’s involvement in the case). In this article we do not further evaluate such scenarios.

### 3.4. Data Requirements for Process Mining Approaches

All mainstream process mining algorithms require case IDs and activities to be recorded accurately in the log. Also, most algorithms require (accurate) timestamps. A recent literature review [3] discovered that the following types of process mining analyses were frequently used in healthcare: discovery techniques (which include process discovery as well as organisational mining approaches such as social network mining), conformance checking, process variant analysis, and performance analysis.

- Process discovery techniques usually take as input a multi-set of traces (i.e., ordered sequences of activity labels) and do not require timestamps; however, timestamps are typically used to order events. Most academic process discovery algorithms (implemented in ProM) and some commercial process discovery tools (e.g., Celonis) can discover formal models with concurrency (represented using modeling notations with well-defined semantics, e.g., Petri nets). Such algorithms require that all events in the log refer to cases. On the other hand, most commercial process mining tools (as well as some ProM plugins) convert the log to Directly Follows Graphs (DFG) annotated with frequencies and times, which show how frequently different activities follow each other and average times between them. Such tools then use the annotated DFG to perform process discovery, conformance, and performance analysis. DFG-based tools do not require complete traces and only require that “directly-follows” relations between activities are preserved in the log.
- Most academic process conformance and performance analysis techniques (e.g., alignment-based approaches) use formal models and require that complete traces are recorded in the log; while most commercial tools work with Directly Follows Graphs.
- Organisational mining techniques require resource information to be recorded in the log (in addition to case IDs, activities, and timestamps). Moreover, resource and data attributes can also be required by conformance checking approaches that consider different process perspectives.
- Process variant analysis, which is concerned with comparing process behaviour and performance of different cohorts, often uses case data attributes to distinguish between cohorts.

To comply with strict privacy requirements for healthcare data, one would need to consider *anonymising (1) event log attribute values and (2) atypical process behaviour*. However, many process mining techniques require that healthcare process data is accurate and representative. That is: *(1) all events belong to a particular case*; *(2) attributes that represent case identifiers and activity labels are accurate; and (3) timestamps are reliable and accurate*. Thus, the need to balance the privacy requirements of healthcare data and the utility requirements of process mining techniques is paramount. In the following section, we assess whether existing privacy-preserving data transformation approaches can preserve the attribute values and relations between events as discussed above.

## 4. Anonymising Healthcare Process Data

In this section, we assess the suitability of different data transformation approaches to anonymise sensitive attribute values (Section 4.1) and atypical process behavior (Section 4.2) that can be present in healthcare process data.

### 4.1. Anonymising Sensitive Attribute Values

**Figure 2:**
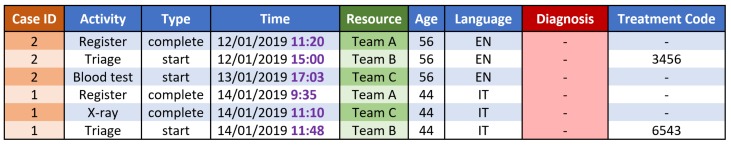
Application of data transformation techniques to the event log in Figure 1: Case ID: swapping; Time: noise addition; Resource: generalisation; Diagnosis: suppression.

As discussed in Section 3, typical event log attributes such as *case, activity, time, resource*, and many data attributes could contribute to identity disclosure. Below, we discuss how these attributes could be anonymised using generic data transformation approaches described in Section 2. We evaluate the suitability of deterministic encryption (referred to here as encryption), which was used to anonymise event log data [9,12], and other traditional data transformation approaches used in the data mining community such as data swapping, value suppression, generalisation, micro-aggregation, and noise addition (which, to the best of our knowledge, were not applied to event logs). Figure 2 depicts how some of these techniques can be applied to the event log in Figure 1.

***Case*** identifiers can be encrypted (as well as other event log attributes); however, encryption does not provide strong data privacy protection (and may not be suitable to protect sensitive healthcare data). An underlying assumption of all process mining algorithms is that case identifiers are unique, which makes the application of value suppression, generalisation, and micro-aggregation not suitable (these methods are used to hide infrequent attribute values). Adding noise to case identifiers can yield values that are no longer unique, which can decrease the accuracy of all process mining algorithms. Data swapping can be applied to case IDs without impact on process mining results.

***Activity*** labels can be encrypted; however, encrypted labels can be identified by someone with knowledge of the process (e.g., most or least frequent activities [12]). Moreover, encryption makes it difficult to interpret analysis results. In addition, one must also encrypt process model labels when applying process mining algorithms that use process models as input (e.g., many process performance and conformance analysis approaches). The application of value suppression, generalisation, and micro-aggregation to activity labels may affect the accuracy of process mining results where the utility loss depends on the process mining algorithm used. For example, removing infrequent activity labels may not have a significant effect on process discovery results (as process models often capture mainstream process behavior); however, process conformance analysis results may become invalid. One can use generalisation or micro-aggregation to hide some sensitive activities (e.g., replace activities “HIV test” and “Hepatitis C test” with activity “Blood test”). The result of process discovery performed on such logs will be correct; however, the discovered process model will be on a higher level of granularity. Noise addition and swapping activity labels will invalidate the results of all process mining algorithms. For example, if activity labels in a log are swapped, the resulting traces will consist of random activity sequences; hence, discovered process models will be incorrect, as well as other process mining results.

***Timestamps*** can be treated as numerical values and encrypted using methods which preserve the order of events. Such encryption will not affect the results of process mining algorithms that work with ordered events and do not require timestamps (such as many process discovery algorithms). On the other hand, an event log with encrypted timestamps will not be suitable for performance analysis. Value suppression, generalisation, and micro-aggregation can be used to anonymise sensitive timestamps (e.g., as discussed in Section 3, according to the HIPAA Privacy Rule admission and discharge times must be anonymised). This will affect the accuracy of most process mining algorithms. For example, if value suppression is applied to admission times, the discovered process model will not include activity “Admission”. On the other hand, if generalisation is applied to admission times (by only leaving the year as prescribed by the HIPAA Privacy Rule), process discovery may not be affected (provided that admission is the first activity in the process and the order of activities is preserved); however, process performance analysis results may become invalid (as the time between admission and other activities in the process will no longer be correct). The effect of micro-aggregation depends on the aggregation operator used to compute a collective value. If the aggregation operator preserves the order of events, then some process mining algorithms may not be affected (e.g., process discovery); however, if the operator changes the order of events (e.g., mean or median), then process mining results may become invalid. Adding noise to timestamps or swapping their values will yield incorrect process mining results (as the order of events in the transformed log is no longer preserved).

***Resource*** information can be encrypted without impacting organisational mining results, while noise addition and swapping will invalidate such results (as resources will no longer be related to correct events and cases). One can apply generalisation or micro-aggregation to resource information (e.g., by replacing individual identifiers with team identifiers), which will yield analysis results on a team level. Value suppression can affect the accuracy of organisational mining techniques (e.g., a discovered social network may have fewer nodes).

***Data*** attributes can be encrypted, though encryption of numerical values can make it difficult to conduct some types of analysis. For example, if *age* is encrypted, one can no longer compare process variants for different age cohorts. Value suppression can decrease the accuracy of process mining algorithms that use data (e.g., when infrequent age values are removed, the corresponding cases will not be included in process variant analysis). Using generalisation and micro-aggregation may decrease the accuracy of conformance analysis methods that consider data; however, it may not have any impact on variant analysis (e.g., when comparing different age groups). Noise addition and data swapping will yield incorrect results for process mining methods that require data.

Application of column suppression to any event log attribute will make it impossible to use process mining algorithms which use this attribute. For example, if resource information is removed, one can no longer analyse the organisational perspective. Row suppression may affect the results of all process mining algorithms—the magnitude of the effect will depend on the number and types of records removed from the data set.

Table 1 summarises the suitability of different data transformation approaches to anonymising event log attribute values. Encryption has a minimal effect on data utility for most process mining algorithms; however, it may not provide a required level of privacy protection. Data swapping can be used to anonymise case IDs; however, the application of this method to other event log attributes will invalidate process mining results. Noise addition will nullify all process mining results. Value suppression, generalisation, and micro-aggregation are not suitable for case IDs (as they have unique values), these methods can be applied to other attributes; however, the accuracy of process mining results may be affected.

### 4.2. Anonymising Atypical Process Behaviour

As discussed in Section 3, relations between events in the log (such as event order or grouping of events by case identifiers) can be used to identify atypical process behaviour (which could be linked to individuals). There could be many different types of atypical process behaviour (e.g., infrequent activity sequences, an abnormal number of resources, or atypical durations). Below, we evaluate three approaches which target anonymisation of atypical process behaviour: a confidentiality framework [12], PRETSA [10], and differential privacy model for event logs [30].

The ***confidentiality framework*** for process mining [12] combines a few data transformation techniques. The first step of the framework is filtering out all cases “that do not reach the minimal frequencies” [12]. The framework changes the structure of an event log: a new attribute “previous activity” is added (which specifies for each event the preceding activity in a case) and case IDs are removed. The transformed event log can be used to extract a DFG. Since events in the transformed log are no longer related to cases, it is impossible to identify traces (and atypical process behaviour). However, the transformed log can no longer be used by process mining algorithms that require complete traces; it is only suitable for DFG-based tools (e.g., commercial process mining tools).

***PRETSA*** [10] is a log sanitisation algorithm, which represents a log as a prefix tree and then transforms the tree until given privacy guarantees are met while striving to preserve directly follows relations. The approach allows anonymising two types of atypical process behaviour: infrequent traces and atypical activity execution times. The article [10] evaluates the impact of the log transformation on the results of process discovery and performance analysis algorithms using three real-life logs including a hospital log. It also compares the performance of PRETSA with a “baseline” approach which filters out infrequent traces. The evaluation showed that PRETSA outperforms the baseline approach on all logs and data utility losses are minimal for event logs which do not have many unique traces. However, for a log in which most traces are unique (a hospital log) the utility of the transformed log is significantly decreased, even more so for stricter privacy requirements (which means that the algorithm may not be suitable for healthcare process data).

The **differential privacy model for event logs** [30] targets process discovery algorithms and supports two types of queries: frequencies of directly follows relations and frequencies of traces (i.e., full activity execution sequences). Noise (Laplacian) is added to the output for privacy protection: higher levels of privacy require more noise. The model was evaluated on two event logs including a hospital log. The results showed that adding noise to directly follows relations frequencies did not have a significant effect on process discovery results for both event logs. On the other hand, adding noise to trace frequencies significantly affected process discovery results for the hospital log.

## 5. Evaluating the Impact of Anonymisation on Process Mining

In this section, we evaluate the impact of some generic data transformation approaches (discussed in Section 4) on the results of process mining methods that are often used in the healthcare domain (discussed in Section 3) for three publicly available healthcare event logs. In Section 5.1, we describe characteristics of the event logs used in the evaluation; in Section 5.2, we discuss anonymisation methods we applied to these logs; in Section 5.3, we present the effects of these methods on various process mining results; and in Section 5.4, we summarise the lessons learned from our evaluation.

### 5.1. Event Logs

In the evaluation, we used three publicly available event logs originating from three hospitals. The first event log, referred to here as the *Sepsis* log (https://data.4tu.nl/repository/uuid:915d2bfb-7e84-49ad-a286-dc35f063a460), contains events related to sepsis cases from a hospital (sepsis is a medical condition caused by an infection), where each case represents a pathway of a patient through the hospital. The timestamps of events in the log were randomised in a way that preserves the time between events within a case. The second event log, referred to here as the *BPIC11* log (https://data.4tu.nl/repository/uuid:d9769f3d-0ab0-4fb8-803b-0d1120ffcf54) (which was used in the First International Business Process Intelligence Challenge (https://www.win.tue.nl/bpi/doku.php?id=2011:challenge)), originates from a Dutch hospital. Cases in this log represent pathways of patients of a Gynaecology department. The third event log, referred to here as the *Billing* log (https://data.4tu.nl/repository/uuid:76c46b83-c930-4798-a1c9-4be94dfeb741), contains events related to the process of billing of medical services provided by a regional hospital. As with the *Sepsis* log, the timestamps in the *Billing* log were randomised while preserving the time between events in a case. Such timestamp randomisation may invalidate the results of process mining methods that analyse changes in process behaviour over time; however, it does not change the results of the process mining methods used in the evaluation (and most other mainstream process mining techniques) as the time between events in cases is preserved.

Table 2 provides information about the characteristics of these three logs. For the *BPIC11* log, we only used events related to cases started during a period of 6 months (1/01/2006–30/06/2006) due to performance issues of many process mining techniques on the complete log (in Table 2 we provide information about the filtered log). Table 2 shows that the event logs have different characteristics, with the number of activities ranging from 16 to 333 and the number of cases ranging from 220 to 100,000. In the *Sepsis* log and the *BPIC11* log most cases follow unique process paths, whereas the *Billing* log is more structured (on average, 98 cases follow a process path).

### 5.2. Anonymisation

In Section 4.1, we assessed the suitability of different generic data transformation approaches to anonymise sensitive attribute values. The analysis showed that encryption has a minimal effect on data utility but provides weak privacy protection; swapping and noise addition invalidate most process mining results; while the application of suppression and generalisation to some attributes can affect process mining results for event logs with certain characteristics. In this section, we conduct an empirical evaluation of the impact of suppression and generalisation on the results of different process mining algorithms applied in the healthcare domain using hospital logs with different characteristics.

As discussed in Section 2, to hide sensitive or infrequent information, one could use suppression, which removes values, rows or columns, or generalisation, which replaces data values with approximate values [13]. In the experiments, we applied generalisation to the timestamp attribute, and suppression was applied to the activity, resource and data attributes. These anonymisation operations target the following privacy threats:

- Activity suppression was used to hide infrequent activities: for a given value of *k* (described below), we suppressed all activities that were not performed in at least *k* cases. Activity suppression targets an infrequent activity linkage threat (e.g., when an adversary with background knowledge of the process can identify a patient by a rare medical test or treatment that was performed in the case).
- Resource suppression was used to hide infrequent resources: for a given value of *k*, we suppressed all resources that were not involved in at least *k* cases. Resource suppression targets an infrequent resource linkage threat (e.g., when an adversary can identify a patient by the involvement in the patient’s case of a doctor who is only involved in exceptional cases).
- Data suppression was used to hide infrequent combinations of case data attributes: for a given value of *k*, we suppressed all combinations of case data attributes that were not associated with at least *k* cases. This anonymisation method targets infrequent case data linkage threat (e.g., when a patient can be identified by a unique combination of case data attributes, e.g., age, language and diagnosis).
- Generalisation was used to replace exact timestamp values with more general values (e.g., by only keeping the year of an event as we describe below). This anonymisation method targets timestamp linkage threat (e.g., when a patient can be identified by their admission time to the hospital).

We created a number of anonymised event logs by applying one anonymisation method to one attribute at a time (the code used to create the anonymised logs and the resulting anonymised logs are uploaded as supplementary materials as described in Appendix A). Please note that each anonymisation method only targets one specific privacy threat (as described above) and does not guarantee privacy protection of personal information in the log (our goal here is to evaluate the impact of each method on process mining results). Below, we provide a detailed description of how different attributes in the three event logs were anonymised.

Suppression was applied to activity labels: events related to a given activity are removed if the activity was not performed in at least *k* cases. This was repeated for all activities for three different values of *k*: k=2, k=10, and k=100. Timestamps were generalised with two levels of granularity: *month*—month and year is kept for each event (while information about date and time is removed); and *year*—only year is kept for each event. In Section 3, we described a requirement of the HIPAA Privacy Rule which prescribes removal of all elements of dates except year related to an individual, including admission and discharge dates. To show the effect of this anonymisation approach, we created a version of the *Sepsis* log in which we only generalised the timestamps of admission and discharge events (the timestamps of other events were not changed). This anonymisation approach was not applied to the other two logs as the *Billing* log does not include patients’ admission and discharge times, and it is not clear which events refer to admission and discharge times in the *BPIC11* log. Please note that events in cases are ordered by timestamps in these logs. Generalisation of all timestamps did not change the order of events; however, generalisation of admission and discharge times resulted in the changed order of events in some cases.

Table 3 shows the percentage of cases and events affected by these privacy-preserving transformations in the three event logs. We can see that few events were affected by activity suppression in the *Billing* log and the *Sepsis* log, while more events were affected in the *BPIC11* log (the log has many activity labels). Generalisation of all timestamps affected all events in the three logs, and generalisation of admission and discharge times in the *Sepsis* log affected 21% of events.

The three event logs include information about resources (employees or organisational groups) who performed process activities. We anonymised resource information in the *Sepsis* log and the *BPIC11* log; the *Billing* log was not used as resource information is missing for many events in the log (44.82%), and we would like to separate data quality issues from the effect of privacy-preserving transformations. The *Sepsis* log and the *BPIC11* log include information about organisational groups (here referred to as resources) responsible for process activities. For each log, we applied value suppression to the resource attribute: a given value of the resource attribute is removed if the resource was not involved in at least *k* cases (for k=2, k=10, and k=100). We also created the second version of the anonymised logs by applying row suppression: an event is removed if the value of its resource attribute is not associated with at least *k* cases (for k=2, k=10, and k=100).

The three event logs have many data attributes; however, most of them have many missing values. To show the effect of anonymisation of data attributes, we used selected data attributes (which do not have any missing values) in the *Sepsis* log. The *Sepsis* log includes a number of data attributes with information related to diagnosis and treatment. We used 22 data attributes recorded for each case that did not have any missing values (5 data attributes with missing values were removed). These 22 data attributes have two values (*true* or *false*). We applied value suppression to these attributes: if a given combination of values of the 22 data attributes is associated with fewer than *k* cases, then the values are suppressed (for k=2, k=10, and k=100).

Table 4 shows the percentage of cases and events in the *Sepsis* log and the *BPIC11* log that were affected by the resource and data anonymisation methods (resource suppression refers to both value suppression and row suppression of the resource attribute—the number of the affected events and cases is the same for both anonymisation methods).

### 5.3. Results

In this section, we use the anonymised event logs (described in Section 5.2) and show how the anonymisation methods affect the results of process mining approaches that are frequently used in the healthcare domain (discussed in Section 3): process discovery, process conformance analysis, process performance analysis, organisational mining, and process variant analysis. For each process mining category, we selected a well-known process mining method implemented as a plugin of the open source process mining framework ProM (http://www.promtools.org/) (version 6.8). Process discovery, process conformance, and performance analysis were conducted for the logs with anonymised timestamps and activity labels; organisational mining was performed for the logs with anonymised resources; and process variant analysis was conducted for the logs with anonymised data attributes.

#### 5.3.1. Process Discovery

We used the ProM plugin “Inductive visual Miner” [32] (the default miner) and discovered process models from the original and the anonymised event logs. For each log, two process models were discovered: (1) a model that captures mainstream process behavior (with paths = 0.8, the default setting of the plugin); and (2) a model that represents all process paths. We then used the projected fitness and precision measures [33] to evaluate the quality of the discovered process models with respect to the original logs. Fitness “expresses the part of the event log that is represented by the model”, while precision measures the “behaviour in the model that is present in the event log” [33].

**Figure 3:**
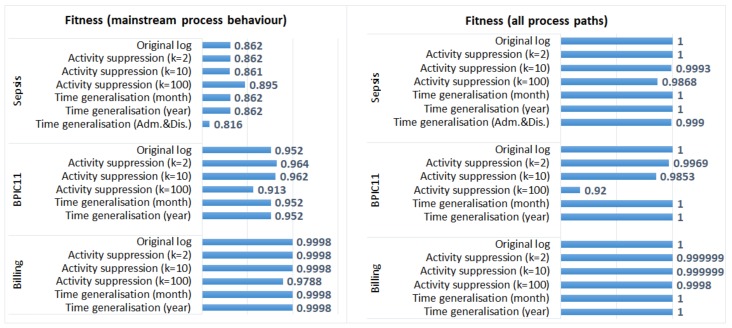
The impact of activity suppression and time generalisation on the results of process discovery: fitness values for the three event logs.

**Figure 4:**
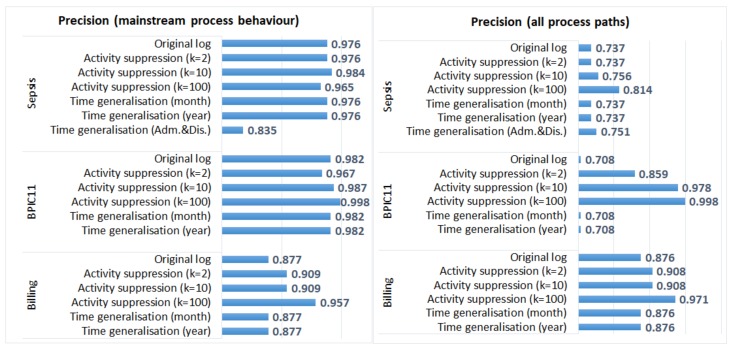
The impact of activity suppression and time generalisation on the results of process discovery: precision values for the three event logs.

Figure 3 shows fitness values and Figure 4 shows precision values for the process models discovered from the original and the anonymised logs. We can see that generalisation of all timestamps did not have any effect on the quality of the discovered models (fitness and precision values of the process models discovered from the original and the anonymised logs are the same). The process discovery plugin we applied uses activity sequences as input and does not require timestamps; therefore, the process models discovered from the logs in which all timestamps were generalised are identical to the models discovered from the corresponding original logs (as discussed in Section 5.2, the order of events was not changed in these anonymised logs). On the other hand, generalisation of admission and discharge timestamps in the *Sepsis* log resulted in the changed order of events in some cases. This affected both fitness and precision values, especially for the process model that represents mainstream process behaviour. Please note that in the evaluation, we use event logs in which events are ordered (by time); if one uses data sets in which events are not already ordered, the effect of anonymisation on process mining results can be different.

The impact of activity suppression for k = 2 and k = 10 is negligible for the *Sepsis* log and the *Billing* log, which is expected as few events were affected by these anonymisation techniques in these logs (see Table 3). Activity suppression for k = 100 affects the quality of process models discovered from all logs; however, the effect is more pronounced for the *BPIC11* log (which has many activity labels and few cases) and is not very significant for the *Billing* log (which has few activity labels and many cases).

#### 5.3.2. Process Conformance Analysis

We applied an alignment-based process conformance analysis plugin [34], which takes as input a normative process model (which specifies the expected process behaviour) and an event log, aligns the log and the model (by relating events in the log to activities in the model), and provides the average trace fitness value (along with other detailed conformance analysis measures). For each original event log, we discovered a process model that represents mainstream process behaviour using the “Inductive visual Miner” [32] process discovery plugin (with default settings). We then used these process models as input to the conformance analysis plugin.

**Figure 5:**
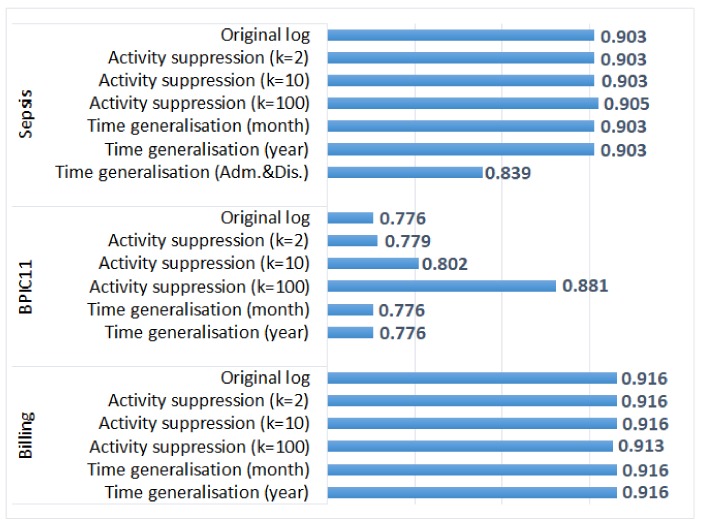
The impact of activity suppression and time generalisation on the results of process conformance analysis: the average trace fitness values for the three event logs.

Figure 5 shows the average trace fitness values for the original and the anonymised event logs. We can see that for the *Sepsis* log and the *Billing* log, the average trace fitness was not affected by most anonymisation methods and was only slightly affected by the application of activity suppression with k = 100. The negligible impact of activity suppression for these two logs can be explained by the fact that few events were affected by the transformation (see Table 3). The average trace fitness was not affected by generalisation of all timestamps as the order of events in cases was not changed. On the other hand, generalisation of admission and discharge timestamps in the *Sepsis* log decreased the average trace fitness value (due to changes in the order of events).

Figure 5 also shows that for the *BPIC11* log, suppressing more infrequent activities improves the average trace fitness value. The process model represents mainstream process behaviour and does not include infrequent activities; therefore, the average trace fitness of the original log is lower than the average trace fitness of the logs with suppressed infrequent activities. Please note that in this experiment, we showed the effect of anonymisation on the average trace fitness; while the average trace fitness may not be changed, fitness values for individual traces in the anonymised logs can be different from fitness values of the corresponding traces in the original logs.

#### 5.3.3. Process Performance Analysis

We used an alignment-based process performance analysis approach [34] implemented as ProM plugin, which takes as input an event log and a process model, aligns the model and the log, and calculates various process performance metrics including the average case throughput time and the average times between activities. For each original log, we discovered a process model that represents all process paths, which was used as input to the plugin.

**Figure 6:**
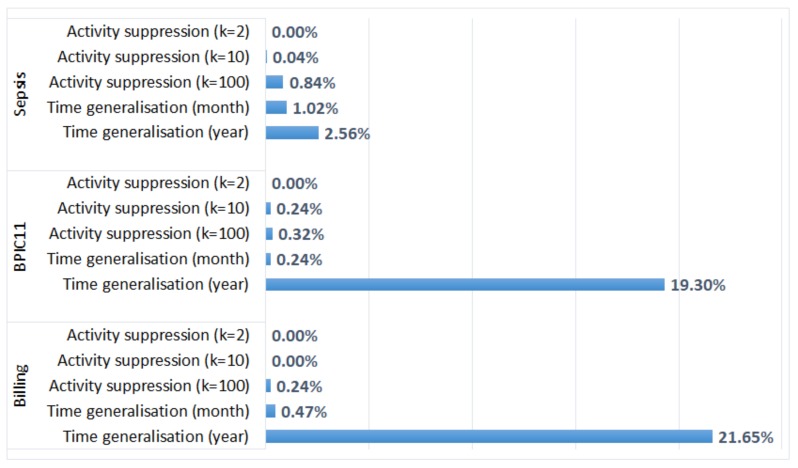
The impact of activity suppression and time generalisation on the results of process performance analysis: the absolute percentage differences between the average case throughput times in the anonymised logs and the corresponding original logs.

For each anonymised log, we report the absolute percentage difference between the average case throughput time in the anonymised log and the corresponding original log. The results are depicted in Figure 6. The figure shows that the average case throughput times for the logs with suppressed activities are not very different from the average case throughput times in the corresponding original logs (the difference is less than 1% for all logs). Time generalisation has a more significant impact on the average case throughput time, especially for the logs in which a timestamp only carries information about the year of an event. For the anonymised *Sepsis* log in which only admission and discharge timestamps were generalised, the absolute percentage difference between the average case throughput times is 645.73% (the value is not shown in Figure 6 due to the large difference with other values). Cases in which the order of events was changed by the transformation may not be aligned with the process model, which affects the process performance analysis results (the plugin only considers cases that are aligned with the model).

**Figure 7:**
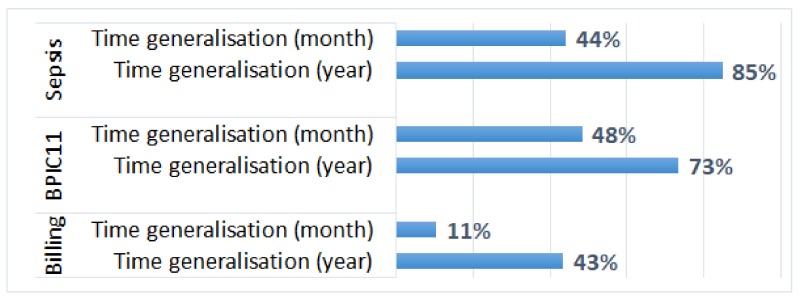
The impact of time generalisation on the results of process performance analysis: the absolute percentage differences between the average times between activities in the original log and the anonymised logs.

We also evaluated the effect of anonymisation on the average times between activities (another process performance metric provided by the plugin). We report the absolute percentage differences between the average times between activities in the original log and the anonymised logs. The performance measure is not available for the suppressed activities; and for the remaining activities, the average times between activities are not changed by activity suppression. The effect of time generalisation on the average times between activities is shown in Figure 7. We can see that the effect of time generalisation on the average times between activities (Figure 7) is more pronounced than the effect on the average case throughput time (Figure 6). Figure 7 does not show the value for the anonymised *Sepsis* log in which only admission and discharge timestamps were generalised, which is 379,921%. The huge difference for this log is expected, as generalisation of admission and discharge timestamps significantly changed the times between these two activities and all other activities in the process.

#### 5.3.4. Organisational Mining

We used ProM plugin “Mine for a Handover-of-Work Social Network” [35], which takes as input an event log and discovers a social network. Nodes in the social network represent resources and the weight of an arc between two nodes is determined by the frequency of activity handovers between these two resources. We discovered social networks from the original logs and from the anonymised logs (with suppressed resources) and compared the discovered social networks.

**Figure 8:**
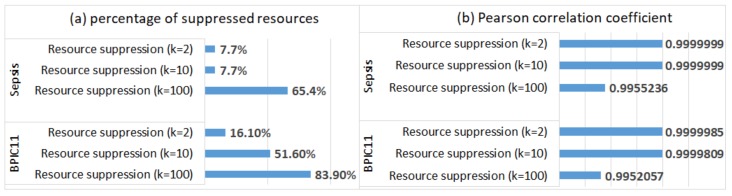
The impact of resource suppression on the results of social network discovery: (**a**) the percentage of suppressed resources, and (**b**) similarity between the arc weights in the social networks discovered from the logs with suppressed events and the corresponding arc weights in the social networks discovered from the original logs (Pearson correlation coefficient).

Social networks discovered from the anonymised logs do not include suppressed resources, and hence, one can no longer analyse activity handovers for these resources (the percentages of the suppressed resources in the logs are shown in Figure 8a). For the logs in which value suppression of the resource attribute was applied, the weights of the arcs between the remaining resources were not changed. This can be explained by the fact that the plugin treats missing resource values as an additional resource (NOT_SET) and creates a corresponding node in the social network; as a result, the weights of the arcs between other resources in the social network are not changed.

For the logs in which event suppression (of the resource attribute) was applied, the weights of the arcs in the discovered social networks were changed. To evaluate the extent of changes, we compared the weights with the corresponding weights in the social networks discovered from the original logs, and we report the Pearson correlation coefficient values in Figure 8b (Pearson coefficient was used to evaluate social network similarity, e.g., in [31]). We can see that the effect of event suppression (of the resource attribute) on the weights of the arcs between the *remaining resources* is negligible, even when most resources are suppressed (for k=100).

#### 5.3.5. Process Variant Analysis

Process variant analysis often involves the comparison of process behavior and performance of different process variants. To compare the performance of different process variants in the *Sepsis* log, we used the alignment-based process performance analysis approach [34] (which was used in Section 5.3.3). To compare process behaviour of different process variants in the *Sepsis* log, we used the alignment-based conformance analysis plugin [34] (which was used in Section 5.3.2).

The *Sepsis* log has a data attribute “InfectionSuspected” with value “true” for 848 cases and value “false” for 202 cases. We split the original log and the anonymised logs (with suppressed data values) based on the value of this attribute, and we refer to cases with value “true” as process variant 1 and to cases with value “false” as process variant 2. Sepsis is a medical condition caused by an infection, and processing of Sepsis cases in which an infection is suspected may be different from processing of cases in which an infection is unknown.

**Figure 9:**
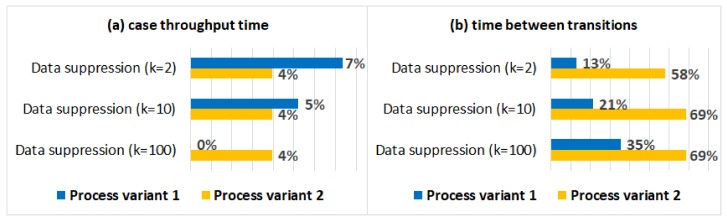
The impact of data suppression on the performance of different process variants: (**a**) the absolute percentage differences between the average case throughput time in the original log and the anonymised logs, and (**b**) the absolute percentage differences between the average times between activities in the original log and the anonymised logs (for process variant 1 and process variant 2).

Figure 9a shows the absolute percentage differences between the average case throughput times in the anonymised logs and the original log (for both process variants). Figure 9b shows the absolute percentage differences between the average times between activities in the original log and the anonymised logs. We can see that the impact of data suppression on the average case throughput time is minimal, while the impact on the average times between activities is significant, especially for process variant 2. As it is shown in Table 4, many cases were affected by data suppression (75% for k=100), which can explain the significant impact on the average times between activities.

**Figure 10:**
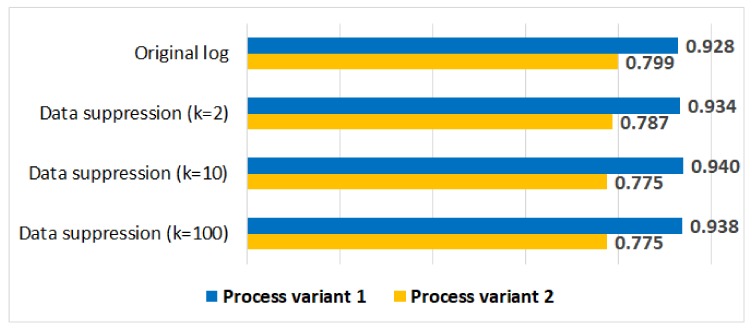
The impact of data suppression on the average trace fitness values for process variant 1 and process variant 2.

Figure 10 shows the average trace fitness values for the original log and for the anonymised logs. We can see that the impact of data suppression on the average trace fitness values is minimal for both process variants.

### 5.4. Discussion

The evaluation results presented in Section 5.3 demonstrated that the impact of an anonymisation method varies for different process mining algorithms and depends on the required privacy level (e.g., the value of parameter *k*) and characteristics of the log. For example, we could see that:

- Generalisation of all timestamps (which did not change the order of events) did not have any effect on the results of process discovery and process conformance analysis plugins that take as input activity sequences (and do not require timestamps); however, it affected the results of process performance analysis;
- Activity suppression, on the other hand, had a minimal effect on the average case throughput time (as start and end activities occur very frequently, and hence, are not suppressed); however, affected process discovery and conformance analysis results in some logs;
- Activity suppression affected many events in the *BPIC11* log (which has many activity labels and few cases) and few events in the other logs (which have few labels and many cases);
- Smaller values of parameter *k* (used in suppression) had a minimal effect on process mining results, while larger values affected the results of some algorithms for some logs (e.g., the results of process conformance analysis for the *BPIC11* log, Figure 5).

Recording the history of privacy-preserving transformations could help to interpret and improve the accuracy of process mining results. For example, if one knows that timestamps were generalised without changing the order of events, then one can trust the results of process discovery algorithms that take as input activity sequences. If a log contains information about events affected by a given privacy-preserving method, then process mining algorithms could use this information to quantify the impact of the method (e.g., by highlighting parts of the model that are less trustworthy). We present a privacy-preserving process mining framework which uses the history of privacy-preserving transformations recorded in privacy metadata in Section 6 and we discuss the proposed privacy metadata for event logs in Section 7.

A limitation of the evaluation presented in this section is the application of versions of some process mining techniques (the conformance analysis plugin [34] and the projected fitness and precision measures [33]) that are optimised for large data sets at the expense of accuracy (due to performance issues of methods that can guarantee the accuracy of results). In the evaluation, we showed the impact of generalisation and suppression (which were selected based on the results of the analysis presented in Section 4) on the results of selected process mining algorithms (which are frequently used in the healthcare domain) for three publicly available hospital logs with different characteristics. Further evaluation could be conducted for other anonymisation techniques and process mining algorithms using event logs originating from different healthcare processes. In this section, we focused on the impact of one anonymisation method applied to one event log attribute. A direction for future work is an evaluation of the impact of anonymisation of multiple attributes and different combinations of privacy-preserving methods.

## 6. Privacy-Preserving Process Mining Framework

On the one hand, the healthcare sector needs to comply with strict data privacy requirements. On the other hand, healthcare process data often contains many sensitive attributes and highly variable process behaviour that presents additional threats to privacy. Ensuring high levels of privacy protection for such data while also preserving data utility for process mining purposes remains an open challenge for the healthcare domain. The analysis of the suitability of existing data transformation approaches to anonymise healthcare process data (presented in Section 4) highlighted the trade-off between data privacy and utility. The methods that preserve higher data utility for process mining purposes (e.g., encryption) do not provide strong privacy protection. On the other hand, the methods that can satisfy stricter privacy requirements (e.g., value suppression and generalisation) can decrease the accuracy of results. The magnitude of the data utility loss depends on the characteristics of a particular log and varies for different process mining algorithms as demonstrated in Section 5. Furthermore, performing analyses on anonymised process data without understanding *how* the data was transformed can yield unpredictable results.

**Figure 11:**
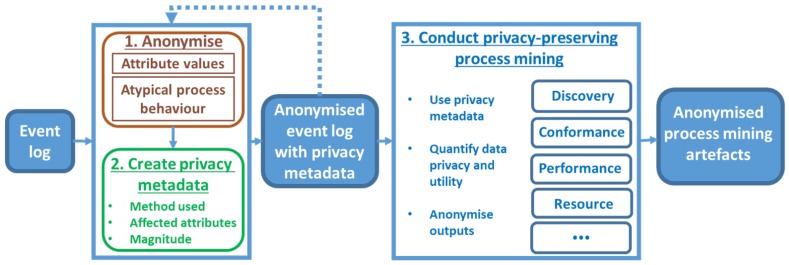
Privacy-preserving process mining framework.

We propose a privacy-preserving process mining framework (Figure 11) which uses a history of privacy-preserving data transformations to quantify their impact and improve the accuracy of process mining results. The framework can be applied to the healthcare domain as well as other domains with high privacy needs. The first two steps of the framework (i.e., data anonymisation and creation of privacy metadata) are performed by the data owner or a trusted party. The third step (i.e., conducting privacy-preserving process mining analysis) can be performed by (not trusted) third parties.

The first step of the framework is ***anonymising*** sensitive information such as sensitive attribute values and atypical process behavior. Anonymisation of sensitive attribute values could be achieved using the data transformation approaches discussed in Section 4.1. Some atypical process behaviours can be anonymised using approaches discussed in Section 4.2; however, methods which could anonymise different types of atypical process behaviour in highly variable processes while preserving data utility for different algorithms are yet to be developed.

The second step of the framework is ***creating privacy metadata***, which maintains the history of privacy-preserving data transformations in a standardised and machine-readable way. The privacy metadata could be used to record information about (1) types of data transformations used (e.g., encryption or generalisation); (2) parts of the log affected by these transformations (e.g., certain attributes or events); (3) the magnitude of the transformations (e.g., by specifying the percentage of affected events); and (4) the reasoning behind the anonymisation used (e.g., an explicit link between legislation and anonymisation actions to show compliance). Such metadata can be stored in an extension to the IEEE XES log format used for process mining and in Section 7 we describe the proposed privacy extension. Please note that one can apply several anonymisation methods, and hence, the first two steps of the framework can be repeated.

The third step of the framework is ***conducting privacy-preserving process mining*** analysis of the anonymised event log with privacy metadata. The privacy metadata can be exploited by new “privacy-aware” process mining techniques to improve mining results. Privacy-aware process mining methods could also quantify data privacy and utility (e.g., by providing confidence measures). The results of process mining techniques could also threaten privacy (by identifying patterns which are linked to individuals). For example, a mined social network could show a much higher number of handovers between two employees, which could reveal their identities to someone with knowledge of the process. There may be a need to further protect process mining outputs (e.g., access control) even after the data itself is anonymised. To the best of our knowledge, anonymisation methods for process mining outputs are yet to be developed. We invite the process mining community to develop novel privacy-aware mining and visualisation techniques by leveraging the proposed privacy metadata.

We presented a general framework that uses privacy metadata to support privacy-preserving process analyses of healthcare process data. The framework supports the selection of anonymisation methods suitable in a given scenario (and does not prescribe the use of specific methods), as different healthcare organisations may have different data privacy and utility requirements which depend on the applicable legislation and the types of analysis one is interested in. The development of tool support for the proposed privacy-preserving process mining framework is a direction for future work. The tool will enable the selection and the application of anonymisation methods and the creation of corresponding privacy metadata (these steps are performed by a trusted party) and the application of privacy-preserving process mining algorithms (this step can be performed by third parties). Data governance issues (such as where how and for how long the data is stored, and who can have access to the data during different stages of its lifecycle) are outside the scope of this work.

## 7. Privacy Metadata

In this section, we describe the proposed privacy extension to the IEEE XES log format (the extension definition in XML format is provided in Appendix B). The privacy extension contains information about privacy-preserving transformations performed on the log. We propose the privacy attributes specified in Table 5 and use the prefix “privacy” for the attributes. A list of (privacy-preserving) **transformations** can be associated with the log, a trace or an event. Each **transformation** is a container with information stored in the following attributes:

- **ID**: the identifier of the anonymisation operation. For example, if one applies activity suppression to the log followed by resource generalisation, then all transformations recorded for the activity suppression will have one ID (e.g., “1”), and transformations recorded for the resource generalisation will have a different ID (e.g., “2”).
- **level**: the attribute is applicable on the log and the trace level and takes one of the two values: “event” (which indicates that the transformation was applied to event attributes) or “trace” (which indicates that the transformation was applied to trace attributes).
- **method**: the applied anonymisation method. Possible values include (but are not limited to): suppression, generalisation, micro-aggregation, swapping, noise addition, encryption.
- **type**: the attribute takes one of the three values: “UPDATE”, “DELETE”, or “INSERT”. Value “UPDATE” is used if the anonymisation method modifies an attribute value (e.g., by adding noise or generalising). Value “DELETE” is used if the anonymisation method removes an attribute value, an event, or a trace. Value “INSERT” is used if the anonymisation method adds a new event or a trace.
- **attributes**: a list of attributes affected by the transformation.
- **attribute**: an attribute affected by the transformation. Value “ALL” indicates that all attributes were affected by the transformation.
- **impact**: the attribute is applicable on the log and on the trace level and specifies the number of traces or events (defined by attribute “level”) affected by the transformation.
- **description**: a list of properties which contain additional information about the transformation.
- **property**: a property with additional information about the transformation. For example, it may be used to specify more details about the anonymisation method (e.g., encryption type), a privacy risk targeted by the transformation (e.g., attribute disclosure) or privacy legislation.

Below, we provide examples of privacy metadata recorded for a log (Listing 1), and for a trace (Listing 2) and an event (Listing 3) in the log, using the XES log format.

Listing 1 shows two transformations applied to the log. In transformation *1* (i.e., the transformation with the value of the “privacy:ID” attribute equal to “1”), values of event attribute “Diagnosis” were suppressed in 230 events in the log and additional information provided in the “privacy:description” list specifies that the transformation targeted the attribute disclosure risk. In the second transformation, case identifiers (trace attribute “concept:name”) were swapped in 1000 traces in the log.

Listing 2 shows two transformations applied to a trace. In transformation 3, event attribute “org:resource” was generalised in 5 events in the trace. In transformation 4, 2 events in the trace were suppressed. Please note that if an event is removed by an anonymisation approach, then the corresponding privacy metadata can be recorded on the trace or on the log level.

Listing 3 provides information about two transformations applied to an event. In transformation 1, suppression was applied to attribute “Diagnosis”. In transformation 5, noise addition was applied to attribute “time:timestamp”. Please note that the first transformation applied to this event has the same identifier (1) as the first transformation recorded for the log (Listing 1) which means that information about diagnosis suppression was recorded on the log and on the event level.

**Listing 1.** An example of log privacy attributes.

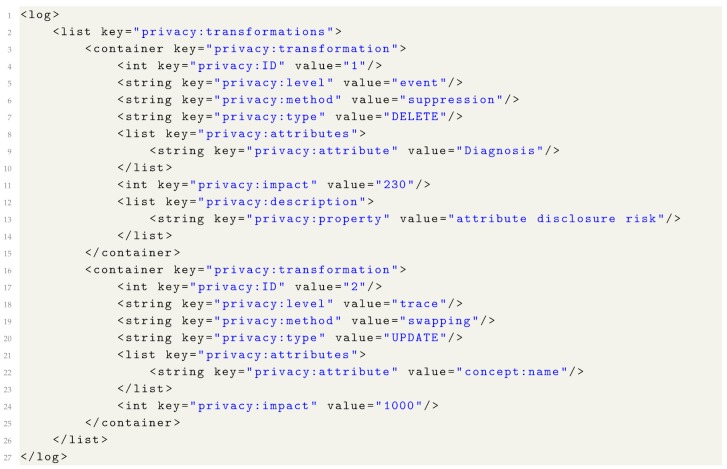



**Listing 2.** An example of trace privacy attributes.

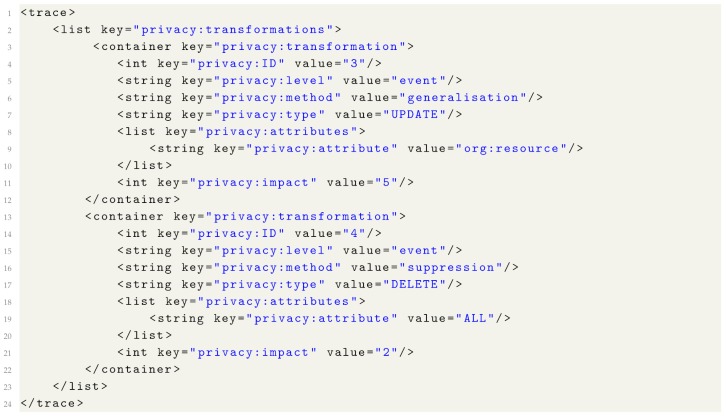



**Listing 3.** An example of event privacy attributes.

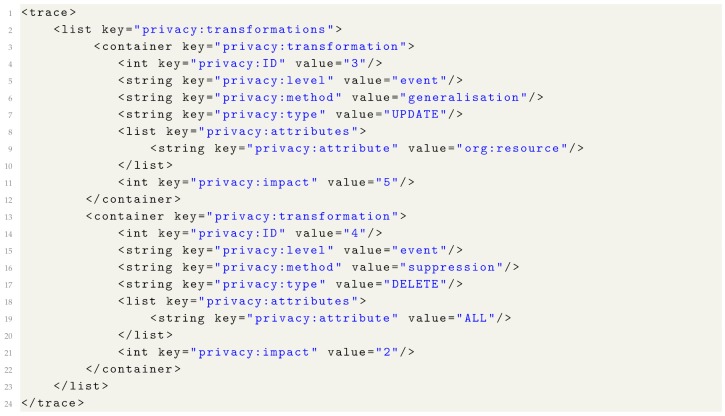



A more detailed history of data transformations can help improve the accuracy of process mining results; however, it may also introduce an avenue for privacy breaches if the metadata can be used to rediscover the original data. Data privacy and utility requirements may vary for different healthcare organisations as they may be subjects to different data privacy regulations. Moreover, data analysis can be performed inside of an organisation, it can be outsourced to a third party, or healthcare data sets can be released for public use – data privacy requirements will be different in these scenarios. To cater for different data privacy and utility needs, the proposed privacy extension allows recording information about privacy-preserving transformations with different levels of detail. For example, one can specify all events that were affected by an anonymisation method or one can specify that the anonymisation affected a given number of events in the log. Such flexibility allows organisations to select a required level of balance of data privacy and utility.

In this section, we presented the first proposal of the privacy extension; we will invite the process mining community to provide feedback and discuss possible enhancements of the proposed extension. A direction for future work is the development of a tool which could support different (existing) log anonymisation methods and annotate the anonymised log with the privacy metadata. Such privacy metadata could be used by process mining algorithms to reason about the extent of changes in the anonymised log, to quantify the impact of privacy-preserving transformations and improve the accuracy of process mining results. For example, a process discovery algorithm could provide a confidence measure for the discovered process model by using information about the number of events in the log which were affected by activity suppression; or a process conformance analysis algorithm could highlight traces affected by anonymisation (and hence, less trustworthy). The development of such privacy-aware process mining algorithms is another direction for future work.

In the presented privacy extension, we focused on recording the history of changes in a log caused by privacy-preserving methods (e.g., generalisation of certain attribute values or suppression of certain events) while preserving data privacy. It is also possible to capture in metadata log characteristics that were not changed by anonymisation. For example, one could record attribute values that were not modified (e.g., certain activity labels) or a list of traces in which the order of events was not altered. Recording such information in a naive way could lead to privacy breaches; for example, if one records a list of attribute values that were not changed by a given privacy-preserving transformation and keeps the history of all privacy-preserving transformations, it may be possible to identify some private attribute values from such metadata. An investigation of privacy-preserving ways to capture unaltered log characteristics is another direction for future work.

## 8. Conclusions

Keeping healthcare process data private while preserving data utility for process mining presents a challenge for the healthcare domain. Until recently, the process mining community did not pay much attention to data privacy issues, while several privacy-preserving data transformation techniques were proposed in the data mining community. However, some of these techniques are not suitable for process data. In this article, we analysed data privacy and utility requirements for healthcare process data and assessed the suitability of existing privacy-preserving data transformation approaches to anonymise such data. We evaluated the effect of some of these anonymisation methods on various process mining results using three publicly available healthcare event logs. The experiments demonstrated that the impact of anonymisation methods varies for different process mining algorithms and depends on the characteristics of a particular log. We proposed a privacy-preserving process mining framework which uses privacy metadata and can support process mining analyses of healthcare processes. Finally, we proposed privacy metadata which records the history of privacy-preserving transformations performed on a log. A direction for future work is the development of tool support for the proposed privacy metadata and the development of privacy-aware process mining algorithms that could leverage the privacy metadata.

## Figures and Tables

**Table 1:** Suitability of privacy-preserving data transformation approaches to anonymising event log attributes: NA: not applicable; `+’: does not affect process mining results; `−’: can be used to anonymise an attribute, however invalidates process mining results; `+/−’: can decrease the accuracy of some process mining methods.

	Case ID	Activity	Time	Resource	Data
Encryption (deterministic)	+	+	+/−	+	+/−
Swapping	+	−	−	−	−
Noise addition	−	−	−	−	−
Value suppression	NA	+/−	+/−	+/−	+/−
Generalisation/micro-aggregation	NA	+/−	+/−	+/−	+/−

**Table 2:** Characteristics of the three healthcare event logs used in the evaluation.

Event Log	Events	Activities	Cases	Process Variants	Cases Per Variant	Log Duration
Sepsis	15,214	16	1050	846	1.2	1 year & 210 days
BPIC11	27,065	333	220	192	1.1	2 years & 88 days
Billing	451,359	18	100,000	1020	98	3 years & 37 days

**Table 3:** The percentage of cases and events affected by the application of activity suppression and time generalisation to the three event logs.

	Sepsis		BPIC11		Billing	
**Anonymisation Method**	**Events** **Affected**	**Cases** **Affected**	**Events** **Affected**	**Cases** **Affected**	**Events** **Affected**	**Cases** **Affected**
Activity suppression (k=2)	0%	0%	0.6%	22%	0.0002%	0.001%
Activity suppression (k=10)	0.04%	0.6%	3%	58%	0.0002%	0.001%
Activity suppression (k=100)	0.7%	10.6%	32%	76%	0.1%	0.2%
Time generalisation, all (month & year)	100%	100%	100%	100%	100%	100%
Time generalisation, Adm.&Dis. (year)	21%	100%	NA	NA	NA	NA

**Table 4:** The percentage of cases and events affected by the application of resource and data anonymisation to the *Sepsis* log and the *BPIC11* log.

	Sepsis		BPIC11	
**Anonymisation Method**	**Events Affected**	**Cases Affected**	**Events Affected**	**Cases Affected**
Resource suppression (k=2)	0.01%	0.2%	0.03%	2.3%
Resource suppression (k=10)	0.01%	0.2%	0.33%	10.5%
Resource suppression (k=100)	5.5%	48.2%	8.3%	65.5%
Data suppression (k=2)	1%	15%	NA	NA
Data suppression (k=10)	2.7%	39%	NA	NA
Data suppression (k=100)	5.2%	75%	NA	NA

**Table 5:** Proposed privacy attributes.

Level	Key	Type	Description
Log, trace, event	transformations	list	A list of applied privacy-preserving transformations.
meta	transformation	container	A container attribute which contains information about each transformation stored in attributes shown below.
meta	ID	int	The identifier of the anonymisation operation.
meta	level	string	Possible values: “trace” or “event” (applicable on the log and on the trace level).
meta	method	string	The applied anonymisation method (e.g., suppression, generalisation, swapping, noise addition, encryption).
meta	type	string	Possible values: “DELETE”, “UPDATE”, or "INSERT".
meta	attributes	list	A list of affected attributes.
meta	attribute	string	An affected attribute; value ‘ALL’ if the transformation affected all attributes.
meta	impact	int	The number of affected items (i.e., traces or events); applicable on the log and on the trace level.
meta	description	list	A list of additional properties of the transformation.
meta	property	string	An additional property of the transformation; for example, encryption type or privacy risk (e.g., identity disclosure).

## Supplementary Materials

The following are available online at https://www.mdpi.com/1660-4601/17/5/1612/s1.

## Appendix A. Anonymisation Code and Anonymised Logs

The anonymised event logs described in Section 5.2 and the code used to create the anonymised logs are uploaded as supplementary materials to the article:

- The anonymisation code (which is also available online (https://bitbucket.org/sbudiono/transformation-tools)) can generalise timestamps with a given level of granularity (e.g., a year or a month) and suppress infrequent values of a given attribute for a given value of *k* (code parameters and examples of usage are described in the `README’ file (https://bitbucket.org/sbudiono/transformation-tools/src/master/README.md)). Values of the parameters used in the experiments are described in Section 5.2.
- All anonymised logs described in Table 3 and Table 4 are uploaded as supplementary materials.

## Appendix B. Privacy Extension Definition

Listing A1 provides a definition of the proposed privacy attributes in XML format, the attributes are discussed in detail in Section 7. Listing A1 specifies the following types of the privacy attributes (standard XES attribute types): *list* attributes (*transformations, attributes, description*), a *container* attribute (*transformation*), *int* attributes (*ID, impact*), and *string* attributes (*level, method, type, attribute, property*).

**Listing A1.** The proposed privacy extension in XML format.
